# Supplementary material for: Optimal minimal residual disease threshold in pediatric acute myeloid leukemia: A retrospective cohort study based on the TARGET database
Source: PLoS Med. 2026 May 8;23(5):e1005088. doi: 10.1371/journal.pmed.1005088 (PMC13155632; doi:10.1371/journal.pmed.1005088)
Supplement: S1 Code — (ZIP) [file pmed.1005088.s002.zip › S2 code/PROJ8_3_tbl1/PROJ8_3_tbl1.htm]

## Kaplan Meier Survival Curve

Outcome: First Event
Time: EFS(years)
Log rank test: implements the G-rho family of Harrington and Fleming (1982), with weights on each death of S(t)^rho, where S is the Kaplan-Meier estimate of survival. With rho = 0 this is the log-rank or Mantel-Haenszel test.

|  |  |  |  |
| --- | --- | --- | --- |
|  | N | Observed | Expected |
| factor(X25.CS2)=0 | 1057 | 483 | 566.2025 |
| factor(X25.CS2)=1 | 16 | 14 | 5.9469 |
| factor(X25.CS2)=2 | 132 | 109 | 33.8506 |

Chisq=
191.8109
on
2
degree of freedom, p=
<0.0001
Survival table

|  |  |  |  |  |  |  |  |
| --- | --- | --- | --- | --- | --- | --- | --- |
|  | EFS(years) | N.Risk | N.Event | N.Censor | Survival | 95%CI Low | 95%CI Upp |
| factor(X25.CS2)=0 | 0.16 | 1057 | 1 | 0 | 0.9991 | 0.9972 | 1.0000 |
| factor(X25.CS2)=0 | 0.17 | 1056 | 2 | 0 | 0.9972 | 0.9940 | 1.0000 |
| factor(X25.CS2)=0 | 0.18 | 1054 | 4 | 0 | 0.9934 | 0.9885 | 0.9983 |
| factor(X25.CS2)=0 | 0.19 | 1050 | 2 | 1 | 0.9915 | 0.9860 | 0.9970 |
| factor(X25.CS2)=0 | 0.2 | 1047 | 5 | 0 | 0.9868 | 0.9799 | 0.9937 |
| factor(X25.CS2)=0 | 0.21 | 1042 | 4 | 0 | 0.9830 | 0.9752 | 0.9908 |
| factor(X25.CS2)=0 | 0.22 | 1038 | 2 | 0 | 0.9811 | 0.9729 | 0.9893 |
| factor(X25.CS2)=0 | 0.23 | 1036 | 2 | 1 | 0.9792 | 0.9706 | 0.9878 |
| factor(X25.CS2)=0 | 0.24 | 1033 | 1 | 0 | 0.9782 | 0.9695 | 0.9871 |
| factor(X25.CS2)=0 | 0.25 | 1032 | 1 | 0 | 0.9773 | 0.9683 | 0.9863 |
| factor(X25.CS2)=0 | 0.26 | 1031 | 4 | 0 | 0.9735 | 0.9638 | 0.9832 |
| factor(X25.CS2)=0 | 0.27 | 1027 | 3 | 1 | 0.9706 | 0.9605 | 0.9809 |
| factor(X25.CS2)=0 | 0.28 | 1023 | 4 | 0 | 0.9668 | 0.9561 | 0.9777 |
| factor(X25.CS2)=0 | 0.29 | 1019 | 3 | 0 | 0.9640 | 0.9528 | 0.9753 |
| factor(X25.CS2)=0 | 0.31 | 1016 | 1 | 0 | 0.9631 | 0.9517 | 0.9745 |
| factor(X25.CS2)=0 | 0.32 | 1015 | 3 | 0 | 0.9602 | 0.9485 | 0.9721 |
| factor(X25.CS2)=0 | 0.33 | 1012 | 3 | 1 | 0.9574 | 0.9452 | 0.9696 |
| factor(X25.CS2)=0 | 0.35 | 1008 | 1 | 0 | 0.9564 | 0.9442 | 0.9688 |
| factor(X25.CS2)=0 | 0.36 | 1007 | 1 | 0 | 0.9555 | 0.9431 | 0.9680 |
| factor(X25.CS2)=0 | 0.37 | 1006 | 3 | 0 | 0.9526 | 0.9399 | 0.9655 |
| factor(X25.CS2)=0 | 0.38 | 1003 | 2 | 0 | 0.9507 | 0.9377 | 0.9639 |
| factor(X25.CS2)=0 | 0.4 | 1001 | 4 | 0 | 0.9469 | 0.9335 | 0.9605 |
| factor(X25.CS2)=0 | 0.41 | 997 | 4 | 0 | 0.9431 | 0.9292 | 0.9572 |
| factor(X25.CS2)=0 | 0.42 | 993 | 3 | 0 | 0.9403 | 0.9261 | 0.9547 |
| factor(X25.CS2)=0 | 0.43 | 990 | 1 | 0 | 0.9393 | 0.9250 | 0.9538 |
| factor(X25.CS2)=0 | 0.44 | 989 | 5 | 0 | 0.9346 | 0.9198 | 0.9496 |
| factor(X25.CS2)=0 | 0.45 | 984 | 3 | 0 | 0.9317 | 0.9166 | 0.9471 |
| factor(X25.CS2)=0 | 0.46 | 981 | 2 | 0 | 0.9298 | 0.9145 | 0.9454 |
| factor(X25.CS2)=0 | 0.47 | 979 | 3 | 0 | 0.9270 | 0.9114 | 0.9428 |
| factor(X25.CS2)=0 | 0.48 | 976 | 2 | 0 | 0.9251 | 0.9093 | 0.9411 |
| factor(X25.CS2)=0 | 0.5 | 974 | 3 | 0 | 0.9222 | 0.9062 | 0.9385 |
| factor(X25.CS2)=0 | 0.51 | 971 | 1 | 0 | 0.9213 | 0.9052 | 0.9377 |
| factor(X25.CS2)=0 | 0.52 | 970 | 5 | 0 | 0.9165 | 0.9000 | 0.9334 |
| factor(X25.CS2)=0 | 0.53 | 965 | 6 | 0 | 0.9108 | 0.8938 | 0.9282 |
| factor(X25.CS2)=0 | 0.54 | 959 | 3 | 0 | 0.9080 | 0.8907 | 0.9256 |
| factor(X25.CS2)=0 | 0.55 | 956 | 4 | 0 | 0.9042 | 0.8866 | 0.9221 |
| factor(X25.CS2)=0 | 0.56 | 952 | 7 | 0 | 0.8975 | 0.8794 | 0.9160 |
| factor(X25.CS2)=0 | 0.57 | 945 | 5 | 0 | 0.8928 | 0.8743 | 0.9117 |
| factor(X25.CS2)=0 | 0.58 | 940 | 5 | 0 | 0.8880 | 0.8692 | 0.9073 |
| factor(X25.CS2)=0 | 0.59 | 935 | 4 | 0 | 0.8842 | 0.8651 | 0.9038 |
| factor(X25.CS2)=0 | 0.6 | 931 | 5 | 0 | 0.8795 | 0.8600 | 0.8994 |
| factor(X25.CS2)=0 | 0.61 | 926 | 1 | 0 | 0.8785 | 0.8590 | 0.8985 |
| factor(X25.CS2)=0 | 0.62 | 925 | 2 | 0 | 0.8766 | 0.8570 | 0.8967 |
| factor(X25.CS2)=0 | 0.63 | 923 | 1 | 0 | 0.8757 | 0.8560 | 0.8958 |
| factor(X25.CS2)=0 | 0.64 | 922 | 2 | 0 | 0.8738 | 0.8540 | 0.8941 |
| factor(X25.CS2)=0 | 0.65 | 920 | 4 | 0 | 0.8700 | 0.8499 | 0.8905 |
| factor(X25.CS2)=0 | 0.66 | 916 | 2 | 0 | 0.8681 | 0.8479 | 0.8888 |
| factor(X25.CS2)=0 | 0.67 | 914 | 3 | 0 | 0.8652 | 0.8449 | 0.8861 |
| factor(X25.CS2)=0 | 0.68 | 911 | 8 | 0 | 0.8576 | 0.8368 | 0.8790 |
| factor(X25.CS2)=0 | 0.69 | 903 | 5 | 0 | 0.8529 | 0.8318 | 0.8745 |
| factor(X25.CS2)=0 | 0.7 | 898 | 5 | 0 | 0.8481 | 0.8267 | 0.8701 |
| factor(X25.CS2)=0 | 0.71 | 893 | 4 | 0 | 0.8443 | 0.8227 | 0.8665 |
| factor(X25.CS2)=0 | 0.72 | 889 | 7 | 0 | 0.8377 | 0.8157 | 0.8603 |
| factor(X25.CS2)=0 | 0.73 | 882 | 4 | 0 | 0.8339 | 0.8117 | 0.8567 |
| factor(X25.CS2)=0 | 0.74 | 878 | 4 | 0 | 0.8301 | 0.8077 | 0.8531 |
| factor(X25.CS2)=0 | 0.75 | 874 | 3 | 0 | 0.8272 | 0.8047 | 0.8504 |
| factor(X25.CS2)=0 | 0.76 | 871 | 10 | 0 | 0.8177 | 0.7948 | 0.8414 |
| factor(X25.CS2)=0 | 0.77 | 861 | 1 | 0 | 0.8168 | 0.7938 | 0.8405 |
| factor(X25.CS2)=0 | 0.78 | 860 | 4 | 1 | 0.8130 | 0.7898 | 0.8369 |
| factor(X25.CS2)=0 | 0.79 | 855 | 8 | 0 | 0.8054 | 0.7818 | 0.8297 |
| factor(X25.CS2)=0 | 0.8 | 847 | 3 | 0 | 0.8025 | 0.7789 | 0.8269 |
| factor(X25.CS2)=0 | 0.81 | 844 | 8 | 0 | 0.7949 | 0.7709 | 0.8197 |
| factor(X25.CS2)=0 | 0.82 | 836 | 9 | 0 | 0.7864 | 0.7620 | 0.8115 |
| factor(X25.CS2)=0 | 0.83 | 827 | 4 | 0 | 0.7826 | 0.7580 | 0.8079 |
| factor(X25.CS2)=0 | 0.84 | 823 | 9 | 0 | 0.7740 | 0.7492 | 0.7997 |
| factor(X25.CS2)=0 | 0.85 | 814 | 2 | 0 | 0.7721 | 0.7472 | 0.7979 |
| factor(X25.CS2)=0 | 0.86 | 812 | 2 | 0 | 0.7702 | 0.7452 | 0.7960 |
| factor(X25.CS2)=0 | 0.87 | 810 | 2 | 0 | 0.7683 | 0.7432 | 0.7942 |
| factor(X25.CS2)=0 | 0.88 | 808 | 8 | 0 | 0.7607 | 0.7354 | 0.7869 |
| factor(X25.CS2)=0 | 0.89 | 800 | 2 | 0 | 0.7588 | 0.7334 | 0.7851 |
| factor(X25.CS2)=0 | 0.9 | 798 | 3 | 0 | 0.7559 | 0.7304 | 0.7823 |
| factor(X25.CS2)=0 | 0.91 | 795 | 5 | 0 | 0.7512 | 0.7255 | 0.7778 |
| factor(X25.CS2)=0 | 0.92 | 790 | 5 | 0 | 0.7464 | 0.7206 | 0.7732 |
| factor(X25.CS2)=0 | 0.93 | 785 | 9 | 0 | 0.7379 | 0.7118 | 0.7649 |
| factor(X25.CS2)=0 | 0.94 | 776 | 2 | 0 | 0.7360 | 0.7098 | 0.7631 |
| factor(X25.CS2)=0 | 0.95 | 774 | 1 | 0 | 0.7350 | 0.7088 | 0.7622 |
| factor(X25.CS2)=0 | 0.96 | 773 | 2 | 1 | 0.7331 | 0.7069 | 0.7603 |
| factor(X25.CS2)=0 | 0.97 | 770 | 3 | 0 | 0.7303 | 0.7039 | 0.7576 |
| factor(X25.CS2)=0 | 0.98 | 767 | 1 | 0 | 0.7293 | 0.7030 | 0.7567 |
| factor(X25.CS2)=0 | 0.99 | 766 | 4 | 1 | 0.7255 | 0.6990 | 0.7530 |
| factor(X25.CS2)=0 | 1 | 761 | 4 | 0 | 0.7217 | 0.6951 | 0.7493 |
| factor(X25.CS2)=0 | 1.01 | 757 | 3 | 0 | 0.7188 | 0.6922 | 0.7465 |
| factor(X25.CS2)=0 | 1.02 | 754 | 3 | 1 | 0.7160 | 0.6892 | 0.7437 |
| factor(X25.CS2)=0 | 1.03 | 750 | 3 | 0 | 0.7131 | 0.6863 | 0.7410 |
| factor(X25.CS2)=0 | 1.05 | 747 | 6 | 0 | 0.7074 | 0.6804 | 0.7354 |
| factor(X25.CS2)=0 | 1.06 | 741 | 3 | 1 | 0.7045 | 0.6775 | 0.7326 |
| factor(X25.CS2)=0 | 1.07 | 737 | 4 | 0 | 0.7007 | 0.6736 | 0.7289 |
| factor(X25.CS2)=0 | 1.08 | 733 | 6 | 0 | 0.6950 | 0.6677 | 0.7233 |
| factor(X25.CS2)=0 | 1.09 | 727 | 2 | 0 | 0.6930 | 0.6657 | 0.7215 |
| factor(X25.CS2)=0 | 1.1 | 725 | 2 | 0 | 0.6911 | 0.6638 | 0.7196 |
| factor(X25.CS2)=0 | 1.11 | 723 | 1 | 2 | 0.6902 | 0.6628 | 0.7187 |
| factor(X25.CS2)=0 | 1.12 | 720 | 3 | 1 | 0.6873 | 0.6598 | 0.7159 |
| factor(X25.CS2)=0 | 1.13 | 716 | 4 | 0 | 0.6835 | 0.6559 | 0.7122 |
| factor(X25.CS2)=0 | 1.14 | 712 | 2 | 1 | 0.6815 | 0.6540 | 0.7103 |
| factor(X25.CS2)=0 | 1.15 | 709 | 1 | 1 | 0.6806 | 0.6530 | 0.7094 |
| factor(X25.CS2)=0 | 1.16 | 707 | 2 | 0 | 0.6787 | 0.6510 | 0.7075 |
| factor(X25.CS2)=0 | 1.17 | 705 | 2 | 0 | 0.6767 | 0.6490 | 0.7056 |
| factor(X25.CS2)=0 | 1.18 | 703 | 2 | 0 | 0.6748 | 0.6471 | 0.7037 |
| factor(X25.CS2)=0 | 1.19 | 701 | 2 | 0 | 0.6729 | 0.6451 | 0.7019 |
| factor(X25.CS2)=0 | 1.2 | 699 | 5 | 0 | 0.6681 | 0.6402 | 0.6972 |
| factor(X25.CS2)=0 | 1.21 | 694 | 1 | 1 | 0.6671 | 0.6392 | 0.6962 |
| factor(X25.CS2)=0 | 1.24 | 692 | 1 | 0 | 0.6661 | 0.6382 | 0.6953 |
| factor(X25.CS2)=0 | 1.25 | 691 | 2 | 1 | 0.6642 | 0.6363 | 0.6934 |
| factor(X25.CS2)=0 | 1.26 | 688 | 1 | 0 | 0.6632 | 0.6353 | 0.6925 |
| factor(X25.CS2)=0 | 1.27 | 687 | 4 | 0 | 0.6594 | 0.6313 | 0.6887 |
| factor(X25.CS2)=0 | 1.28 | 683 | 2 | 1 | 0.6575 | 0.6294 | 0.6868 |
| factor(X25.CS2)=0 | 1.3 | 680 | 4 | 0 | 0.6536 | 0.6254 | 0.6830 |
| factor(X25.CS2)=0 | 1.31 | 676 | 2 | 0 | 0.6517 | 0.6235 | 0.6811 |
| factor(X25.CS2)=0 | 1.32 | 674 | 1 | 0 | 0.6507 | 0.6225 | 0.6802 |
| factor(X25.CS2)=0 | 1.34 | 673 | 3 | 1 | 0.6478 | 0.6195 | 0.6773 |
| factor(X25.CS2)=0 | 1.35 | 669 | 3 | 0 | 0.6449 | 0.6166 | 0.6745 |
| factor(X25.CS2)=0 | 1.36 | 666 | 3 | 0 | 0.6420 | 0.6136 | 0.6717 |
| factor(X25.CS2)=0 | 1.37 | 663 | 2 | 1 | 0.6400 | 0.6116 | 0.6698 |
| factor(X25.CS2)=0 | 1.38 | 660 | 3 | 0 | 0.6371 | 0.6087 | 0.6669 |
| factor(X25.CS2)=0 | 1.39 | 657 | 4 | 1 | 0.6333 | 0.6047 | 0.6631 |
| factor(X25.CS2)=0 | 1.4 | 652 | 1 | 0 | 0.6323 | 0.6038 | 0.6622 |
| factor(X25.CS2)=0 | 1.41 | 651 | 1 | 0 | 0.6313 | 0.6028 | 0.6612 |
| factor(X25.CS2)=0 | 1.42 | 650 | 2 | 0 | 0.6294 | 0.6008 | 0.6593 |
| factor(X25.CS2)=0 | 1.43 | 648 | 4 | 0 | 0.6255 | 0.5968 | 0.6555 |
| factor(X25.CS2)=0 | 1.45 | 644 | 2 | 0 | 0.6235 | 0.5949 | 0.6536 |
| factor(X25.CS2)=0 | 1.46 | 642 | 1 | 0 | 0.6226 | 0.5939 | 0.6526 |
| factor(X25.CS2)=0 | 1.48 | 641 | 2 | 0 | 0.6206 | 0.5919 | 0.6507 |
| factor(X25.CS2)=0 | 1.49 | 639 | 3 | 0 | 0.6177 | 0.5890 | 0.6479 |
| factor(X25.CS2)=0 | 1.5 | 636 | 2 | 0 | 0.6158 | 0.5870 | 0.6460 |
| factor(X25.CS2)=0 | 1.51 | 634 | 1 | 0 | 0.6148 | 0.5860 | 0.6450 |
| factor(X25.CS2)=0 | 1.53 | 633 | 1 | 0 | 0.6138 | 0.5850 | 0.6440 |
| factor(X25.CS2)=0 | 1.54 | 632 | 1 | 0 | 0.6129 | 0.5840 | 0.6431 |
| factor(X25.CS2)=0 | 1.55 | 631 | 3 | 0 | 0.6099 | 0.5811 | 0.6402 |
| factor(X25.CS2)=0 | 1.56 | 628 | 1 | 0 | 0.6090 | 0.5801 | 0.6393 |
| factor(X25.CS2)=0 | 1.58 | 627 | 5 | 0 | 0.6041 | 0.5752 | 0.6345 |
| factor(X25.CS2)=0 | 1.6 | 622 | 2 | 0 | 0.6022 | 0.5732 | 0.6326 |
| factor(X25.CS2)=0 | 1.61 | 620 | 0 | 1 | 0.6022 | 0.5732 | 0.6326 |
| factor(X25.CS2)=0 | 1.62 | 619 | 2 | 0 | 0.6002 | 0.5713 | 0.6307 |
| factor(X25.CS2)=0 | 1.64 | 617 | 1 | 0 | 0.5993 | 0.5703 | 0.6297 |
| factor(X25.CS2)=0 | 1.65 | 616 | 2 | 0 | 0.5973 | 0.5683 | 0.6278 |
| factor(X25.CS2)=0 | 1.66 | 614 | 1 | 0 | 0.5963 | 0.5673 | 0.6268 |
| factor(X25.CS2)=0 | 1.67 | 613 | 1 | 0 | 0.5954 | 0.5663 | 0.6259 |
| factor(X25.CS2)=0 | 1.71 | 612 | 0 | 1 | 0.5954 | 0.5663 | 0.6259 |
| factor(X25.CS2)=0 | 1.72 | 611 | 3 | 0 | 0.5924 | 0.5634 | 0.6230 |
| factor(X25.CS2)=0 | 1.75 | 608 | 0 | 1 | 0.5924 | 0.5634 | 0.6230 |
| factor(X25.CS2)=0 | 1.77 | 607 | 1 | 0 | 0.5915 | 0.5624 | 0.6220 |
| factor(X25.CS2)=0 | 1.78 | 606 | 1 | 0 | 0.5905 | 0.5614 | 0.6211 |
| factor(X25.CS2)=0 | 1.79 | 605 | 1 | 0 | 0.5895 | 0.5604 | 0.6201 |
| factor(X25.CS2)=0 | 1.81 | 604 | 1 | 0 | 0.5885 | 0.5594 | 0.6191 |
| factor(X25.CS2)=0 | 1.83 | 603 | 0 | 1 | 0.5885 | 0.5594 | 0.6191 |
| factor(X25.CS2)=0 | 1.87 | 602 | 1 | 1 | 0.5876 | 0.5585 | 0.6182 |
| factor(X25.CS2)=0 | 1.89 | 600 | 2 | 0 | 0.5856 | 0.5565 | 0.6162 |
| factor(X25.CS2)=0 | 1.9 | 598 | 2 | 0 | 0.5836 | 0.5545 | 0.6143 |
| factor(X25.CS2)=0 | 1.92 | 596 | 2 | 1 | 0.5817 | 0.5525 | 0.6124 |
| factor(X25.CS2)=0 | 1.94 | 593 | 1 | 2 | 0.5807 | 0.5515 | 0.6114 |
| factor(X25.CS2)=0 | 1.96 | 590 | 0 | 1 | 0.5807 | 0.5515 | 0.6114 |
| factor(X25.CS2)=0 | 1.98 | 589 | 2 | 1 | 0.5787 | 0.5495 | 0.6095 |
| factor(X25.CS2)=0 | 1.99 | 586 | 1 | 0 | 0.5777 | 0.5485 | 0.6085 |
| factor(X25.CS2)=0 | 2.03 | 585 | 1 | 0 | 0.5768 | 0.5475 | 0.6075 |
| factor(X25.CS2)=0 | 2.05 | 584 | 1 | 0 | 0.5758 | 0.5465 | 0.6065 |
| factor(X25.CS2)=0 | 2.07 | 583 | 1 | 0 | 0.5748 | 0.5455 | 0.6056 |
| factor(X25.CS2)=0 | 2.08 | 582 | 1 | 0 | 0.5738 | 0.5446 | 0.6046 |
| factor(X25.CS2)=0 | 2.09 | 581 | 2 | 0 | 0.5718 | 0.5426 | 0.6026 |
| factor(X25.CS2)=0 | 2.1 | 579 | 0 | 1 | 0.5718 | 0.5426 | 0.6026 |
| factor(X25.CS2)=0 | 2.11 | 578 | 1 | 0 | 0.5708 | 0.5416 | 0.6017 |
| factor(X25.CS2)=0 | 2.14 | 577 | 1 | 0 | 0.5698 | 0.5406 | 0.6007 |
| factor(X25.CS2)=0 | 2.15 | 576 | 1 | 0 | 0.5688 | 0.5396 | 0.5997 |
| factor(X25.CS2)=0 | 2.18 | 575 | 0 | 1 | 0.5688 | 0.5396 | 0.5997 |
| factor(X25.CS2)=0 | 2.2 | 574 | 1 | 0 | 0.5679 | 0.5386 | 0.5987 |
| factor(X25.CS2)=0 | 2.22 | 573 | 2 | 1 | 0.5659 | 0.5366 | 0.5968 |
| factor(X25.CS2)=0 | 2.23 | 570 | 1 | 1 | 0.5649 | 0.5356 | 0.5958 |
| factor(X25.CS2)=0 | 2.27 | 568 | 0 | 2 | 0.5649 | 0.5356 | 0.5958 |
| factor(X25.CS2)=0 | 2.3 | 566 | 1 | 0 | 0.5639 | 0.5346 | 0.5948 |
| factor(X25.CS2)=0 | 2.31 | 565 | 1 | 0 | 0.5629 | 0.5336 | 0.5938 |
| factor(X25.CS2)=0 | 2.32 | 564 | 0 | 2 | 0.5629 | 0.5336 | 0.5938 |
| factor(X25.CS2)=0 | 2.34 | 562 | 1 | 0 | 0.5619 | 0.5325 | 0.5928 |
| factor(X25.CS2)=0 | 2.36 | 561 | 1 | 0 | 0.5609 | 0.5315 | 0.5919 |
| factor(X25.CS2)=0 | 2.37 | 560 | 1 | 1 | 0.5599 | 0.5305 | 0.5909 |
| factor(X25.CS2)=0 | 2.39 | 558 | 0 | 1 | 0.5599 | 0.5305 | 0.5909 |
| factor(X25.CS2)=0 | 2.4 | 557 | 0 | 1 | 0.5599 | 0.5305 | 0.5909 |
| factor(X25.CS2)=0 | 2.41 | 556 | 0 | 1 | 0.5599 | 0.5305 | 0.5909 |
| factor(X25.CS2)=0 | 2.42 | 555 | 1 | 0 | 0.5589 | 0.5295 | 0.5899 |
| factor(X25.CS2)=0 | 2.43 | 554 | 0 | 1 | 0.5589 | 0.5295 | 0.5899 |
| factor(X25.CS2)=0 | 2.44 | 553 | 0 | 2 | 0.5589 | 0.5295 | 0.5899 |
| factor(X25.CS2)=0 | 2.46 | 551 | 0 | 1 | 0.5589 | 0.5295 | 0.5899 |
| factor(X25.CS2)=0 | 2.47 | 550 | 0 | 1 | 0.5589 | 0.5295 | 0.5899 |
| factor(X25.CS2)=0 | 2.48 | 549 | 1 | 0 | 0.5579 | 0.5285 | 0.5889 |
| factor(X25.CS2)=0 | 2.49 | 548 | 0 | 1 | 0.5579 | 0.5285 | 0.5889 |
| factor(X25.CS2)=0 | 2.5 | 547 | 0 | 4 | 0.5579 | 0.5285 | 0.5889 |
| factor(X25.CS2)=0 | 2.51 | 543 | 1 | 1 | 0.5568 | 0.5274 | 0.5878 |
| factor(X25.CS2)=0 | 2.52 | 541 | 1 | 0 | 0.5558 | 0.5264 | 0.5868 |
| factor(X25.CS2)=0 | 2.53 | 540 | 1 | 1 | 0.5548 | 0.5254 | 0.5858 |
| factor(X25.CS2)=0 | 2.55 | 538 | 0 | 1 | 0.5548 | 0.5254 | 0.5858 |
| factor(X25.CS2)=0 | 2.56 | 537 | 0 | 1 | 0.5548 | 0.5254 | 0.5858 |
| factor(X25.CS2)=0 | 2.6 | 536 | 0 | 1 | 0.5548 | 0.5254 | 0.5858 |
| factor(X25.CS2)=0 | 2.61 | 535 | 1 | 1 | 0.5537 | 0.5243 | 0.5848 |
| factor(X25.CS2)=0 | 2.62 | 533 | 2 | 0 | 0.5517 | 0.5222 | 0.5827 |
| factor(X25.CS2)=0 | 2.63 | 531 | 0 | 1 | 0.5517 | 0.5222 | 0.5827 |
| factor(X25.CS2)=0 | 2.64 | 530 | 0 | 1 | 0.5517 | 0.5222 | 0.5827 |
| factor(X25.CS2)=0 | 2.68 | 529 | 0 | 1 | 0.5517 | 0.5222 | 0.5827 |
| factor(X25.CS2)=0 | 2.7 | 528 | 1 | 1 | 0.5506 | 0.5212 | 0.5817 |
| factor(X25.CS2)=0 | 2.71 | 526 | 0 | 1 | 0.5506 | 0.5212 | 0.5817 |
| factor(X25.CS2)=0 | 2.73 | 525 | 0 | 1 | 0.5506 | 0.5212 | 0.5817 |
| factor(X25.CS2)=0 | 2.75 | 524 | 0 | 1 | 0.5506 | 0.5212 | 0.5817 |
| factor(X25.CS2)=0 | 2.78 | 523 | 0 | 2 | 0.5506 | 0.5212 | 0.5817 |
| factor(X25.CS2)=0 | 2.79 | 521 | 0 | 2 | 0.5506 | 0.5212 | 0.5817 |
| factor(X25.CS2)=0 | 2.83 | 519 | 1 | 1 | 0.5495 | 0.5201 | 0.5807 |
| factor(X25.CS2)=0 | 2.89 | 517 | 0 | 1 | 0.5495 | 0.5201 | 0.5807 |
| factor(X25.CS2)=0 | 2.93 | 516 | 0 | 1 | 0.5495 | 0.5201 | 0.5807 |
| factor(X25.CS2)=0 | 2.95 | 515 | 0 | 1 | 0.5495 | 0.5201 | 0.5807 |
| factor(X25.CS2)=0 | 2.98 | 514 | 0 | 1 | 0.5495 | 0.5201 | 0.5807 |
| factor(X25.CS2)=0 | 2.99 | 513 | 1 | 0 | 0.5485 | 0.5190 | 0.5796 |
| factor(X25.CS2)=0 | 3 | 512 | 0 | 1 | 0.5485 | 0.5190 | 0.5796 |
| factor(X25.CS2)=0 | 3.01 | 511 | 0 | 1 | 0.5485 | 0.5190 | 0.5796 |
| factor(X25.CS2)=0 | 3.02 | 510 | 0 | 1 | 0.5485 | 0.5190 | 0.5796 |
| factor(X25.CS2)=0 | 3.03 | 509 | 0 | 1 | 0.5485 | 0.5190 | 0.5796 |
| factor(X25.CS2)=0 | 3.04 | 508 | 0 | 2 | 0.5485 | 0.5190 | 0.5796 |
| factor(X25.CS2)=0 | 3.05 | 506 | 0 | 2 | 0.5485 | 0.5190 | 0.5796 |
| factor(X25.CS2)=0 | 3.07 | 504 | 0 | 1 | 0.5485 | 0.5190 | 0.5796 |
| factor(X25.CS2)=0 | 3.11 | 503 | 0 | 1 | 0.5485 | 0.5190 | 0.5796 |
| factor(X25.CS2)=0 | 3.14 | 502 | 0 | 1 | 0.5485 | 0.5190 | 0.5796 |
| factor(X25.CS2)=0 | 3.15 | 501 | 0 | 1 | 0.5485 | 0.5190 | 0.5796 |
| factor(X25.CS2)=0 | 3.16 | 500 | 0 | 2 | 0.5485 | 0.5190 | 0.5796 |
| factor(X25.CS2)=0 | 3.17 | 498 | 0 | 1 | 0.5485 | 0.5190 | 0.5796 |
| factor(X25.CS2)=0 | 3.18 | 497 | 1 | 3 | 0.5474 | 0.5179 | 0.5785 |
| factor(X25.CS2)=0 | 3.19 | 493 | 0 | 1 | 0.5474 | 0.5179 | 0.5785 |
| factor(X25.CS2)=0 | 3.2 | 492 | 0 | 1 | 0.5474 | 0.5179 | 0.5785 |
| factor(X25.CS2)=0 | 3.22 | 491 | 0 | 1 | 0.5474 | 0.5179 | 0.5785 |
| factor(X25.CS2)=0 | 3.23 | 490 | 0 | 1 | 0.5474 | 0.5179 | 0.5785 |
| factor(X25.CS2)=0 | 3.24 | 489 | 0 | 1 | 0.5474 | 0.5179 | 0.5785 |
| factor(X25.CS2)=0 | 3.25 | 488 | 0 | 2 | 0.5474 | 0.5179 | 0.5785 |
| factor(X25.CS2)=0 | 3.28 | 486 | 0 | 3 | 0.5474 | 0.5179 | 0.5785 |
| factor(X25.CS2)=0 | 3.29 | 483 | 0 | 1 | 0.5474 | 0.5179 | 0.5785 |
| factor(X25.CS2)=0 | 3.3 | 482 | 0 | 2 | 0.5474 | 0.5179 | 0.5785 |
| factor(X25.CS2)=0 | 3.32 | 480 | 0 | 1 | 0.5474 | 0.5179 | 0.5785 |
| factor(X25.CS2)=0 | 3.33 | 479 | 0 | 3 | 0.5474 | 0.5179 | 0.5785 |
| factor(X25.CS2)=0 | 3.34 | 476 | 0 | 2 | 0.5474 | 0.5179 | 0.5785 |
| factor(X25.CS2)=0 | 3.35 | 474 | 0 | 3 | 0.5474 | 0.5179 | 0.5785 |
| factor(X25.CS2)=0 | 3.36 | 471 | 0 | 2 | 0.5474 | 0.5179 | 0.5785 |
| factor(X25.CS2)=0 | 3.38 | 469 | 0 | 3 | 0.5474 | 0.5179 | 0.5785 |
| factor(X25.CS2)=0 | 3.39 | 466 | 1 | 2 | 0.5462 | 0.5167 | 0.5774 |
| factor(X25.CS2)=0 | 3.4 | 463 | 0 | 1 | 0.5462 | 0.5167 | 0.5774 |
| factor(X25.CS2)=0 | 3.41 | 462 | 0 | 4 | 0.5462 | 0.5167 | 0.5774 |
| factor(X25.CS2)=0 | 3.43 | 458 | 0 | 1 | 0.5462 | 0.5167 | 0.5774 |
| factor(X25.CS2)=0 | 3.44 | 457 | 0 | 4 | 0.5462 | 0.5167 | 0.5774 |
| factor(X25.CS2)=0 | 3.45 | 453 | 0 | 2 | 0.5462 | 0.5167 | 0.5774 |
| factor(X25.CS2)=0 | 3.46 | 451 | 0 | 1 | 0.5462 | 0.5167 | 0.5774 |
| factor(X25.CS2)=0 | 3.48 | 450 | 0 | 2 | 0.5462 | 0.5167 | 0.5774 |
| factor(X25.CS2)=0 | 3.5 | 448 | 0 | 3 | 0.5462 | 0.5167 | 0.5774 |
| factor(X25.CS2)=0 | 3.52 | 445 | 0 | 1 | 0.5462 | 0.5167 | 0.5774 |
| factor(X25.CS2)=0 | 3.53 | 444 | 0 | 1 | 0.5462 | 0.5167 | 0.5774 |
| factor(X25.CS2)=0 | 3.54 | 443 | 1 | 1 | 0.5450 | 0.5154 | 0.5762 |
| factor(X25.CS2)=0 | 3.56 | 441 | 0 | 4 | 0.5450 | 0.5154 | 0.5762 |
| factor(X25.CS2)=0 | 3.58 | 437 | 0 | 1 | 0.5450 | 0.5154 | 0.5762 |
| factor(X25.CS2)=0 | 3.62 | 436 | 0 | 1 | 0.5450 | 0.5154 | 0.5762 |
| factor(X25.CS2)=0 | 3.63 | 435 | 0 | 1 | 0.5450 | 0.5154 | 0.5762 |
| factor(X25.CS2)=0 | 3.65 | 434 | 0 | 2 | 0.5450 | 0.5154 | 0.5762 |
| factor(X25.CS2)=0 | 3.66 | 432 | 0 | 1 | 0.5450 | 0.5154 | 0.5762 |
| factor(X25.CS2)=0 | 3.74 | 431 | 0 | 1 | 0.5450 | 0.5154 | 0.5762 |
| factor(X25.CS2)=0 | 3.79 | 430 | 0 | 1 | 0.5450 | 0.5154 | 0.5762 |
| factor(X25.CS2)=0 | 3.81 | 429 | 0 | 2 | 0.5450 | 0.5154 | 0.5762 |
| factor(X25.CS2)=0 | 3.83 | 427 | 0 | 1 | 0.5450 | 0.5154 | 0.5762 |
| factor(X25.CS2)=0 | 3.84 | 426 | 0 | 2 | 0.5450 | 0.5154 | 0.5762 |
| factor(X25.CS2)=0 | 3.87 | 424 | 0 | 1 | 0.5450 | 0.5154 | 0.5762 |
| factor(X25.CS2)=0 | 3.88 | 423 | 0 | 1 | 0.5450 | 0.5154 | 0.5762 |
| factor(X25.CS2)=0 | 3.89 | 422 | 0 | 1 | 0.5450 | 0.5154 | 0.5762 |
| factor(X25.CS2)=0 | 3.9 | 421 | 0 | 2 | 0.5450 | 0.5154 | 0.5762 |
| factor(X25.CS2)=0 | 3.95 | 419 | 0 | 1 | 0.5450 | 0.5154 | 0.5762 |
| factor(X25.CS2)=0 | 3.96 | 418 | 0 | 1 | 0.5450 | 0.5154 | 0.5762 |
| factor(X25.CS2)=0 | 3.97 | 417 | 0 | 1 | 0.5450 | 0.5154 | 0.5762 |
| factor(X25.CS2)=0 | 3.99 | 416 | 1 | 0 | 0.5437 | 0.5141 | 0.5749 |
| factor(X25.CS2)=0 | 4.04 | 415 | 0 | 2 | 0.5437 | 0.5141 | 0.5749 |
| factor(X25.CS2)=0 | 4.05 | 413 | 0 | 2 | 0.5437 | 0.5141 | 0.5749 |
| factor(X25.CS2)=0 | 4.06 | 411 | 0 | 2 | 0.5437 | 0.5141 | 0.5749 |
| factor(X25.CS2)=0 | 4.07 | 409 | 1 | 1 | 0.5423 | 0.5127 | 0.5736 |
| factor(X25.CS2)=0 | 4.08 | 407 | 0 | 2 | 0.5423 | 0.5127 | 0.5736 |
| factor(X25.CS2)=0 | 4.13 | 405 | 0 | 2 | 0.5423 | 0.5127 | 0.5736 |
| factor(X25.CS2)=0 | 4.15 | 403 | 0 | 2 | 0.5423 | 0.5127 | 0.5736 |
| factor(X25.CS2)=0 | 4.16 | 401 | 0 | 1 | 0.5423 | 0.5127 | 0.5736 |
| factor(X25.CS2)=0 | 4.19 | 400 | 0 | 2 | 0.5423 | 0.5127 | 0.5736 |
| factor(X25.CS2)=0 | 4.22 | 398 | 0 | 2 | 0.5423 | 0.5127 | 0.5736 |
| factor(X25.CS2)=0 | 4.23 | 396 | 1 | 0 | 0.5410 | 0.5113 | 0.5723 |
| factor(X25.CS2)=0 | 4.26 | 395 | 0 | 1 | 0.5410 | 0.5113 | 0.5723 |
| factor(X25.CS2)=0 | 4.27 | 394 | 0 | 2 | 0.5410 | 0.5113 | 0.5723 |
| factor(X25.CS2)=0 | 4.31 | 392 | 0 | 2 | 0.5410 | 0.5113 | 0.5723 |
| factor(X25.CS2)=0 | 4.32 | 390 | 0 | 1 | 0.5410 | 0.5113 | 0.5723 |
| factor(X25.CS2)=0 | 4.33 | 389 | 0 | 1 | 0.5410 | 0.5113 | 0.5723 |
| factor(X25.CS2)=0 | 4.34 | 388 | 0 | 1 | 0.5410 | 0.5113 | 0.5723 |
| factor(X25.CS2)=0 | 4.35 | 387 | 0 | 4 | 0.5410 | 0.5113 | 0.5723 |
| factor(X25.CS2)=0 | 4.36 | 383 | 0 | 4 | 0.5410 | 0.5113 | 0.5723 |
| factor(X25.CS2)=0 | 4.38 | 379 | 0 | 3 | 0.5410 | 0.5113 | 0.5723 |
| factor(X25.CS2)=0 | 4.4 | 376 | 0 | 1 | 0.5410 | 0.5113 | 0.5723 |
| factor(X25.CS2)=0 | 4.41 | 375 | 0 | 2 | 0.5410 | 0.5113 | 0.5723 |
| factor(X25.CS2)=0 | 4.42 | 373 | 0 | 2 | 0.5410 | 0.5113 | 0.5723 |
| factor(X25.CS2)=0 | 4.44 | 371 | 0 | 3 | 0.5410 | 0.5113 | 0.5723 |
| factor(X25.CS2)=0 | 4.45 | 368 | 0 | 1 | 0.5410 | 0.5113 | 0.5723 |
| factor(X25.CS2)=0 | 4.47 | 367 | 0 | 2 | 0.5410 | 0.5113 | 0.5723 |
| factor(X25.CS2)=0 | 4.48 | 365 | 0 | 2 | 0.5410 | 0.5113 | 0.5723 |
| factor(X25.CS2)=0 | 4.49 | 363 | 0 | 2 | 0.5410 | 0.5113 | 0.5723 |
| factor(X25.CS2)=0 | 4.5 | 361 | 0 | 1 | 0.5410 | 0.5113 | 0.5723 |
| factor(X25.CS2)=0 | 4.53 | 360 | 0 | 3 | 0.5410 | 0.5113 | 0.5723 |
| factor(X25.CS2)=0 | 4.54 | 357 | 0 | 1 | 0.5410 | 0.5113 | 0.5723 |
| factor(X25.CS2)=0 | 4.55 | 356 | 0 | 2 | 0.5410 | 0.5113 | 0.5723 |
| factor(X25.CS2)=0 | 4.58 | 354 | 0 | 2 | 0.5410 | 0.5113 | 0.5723 |
| factor(X25.CS2)=0 | 4.59 | 352 | 0 | 1 | 0.5410 | 0.5113 | 0.5723 |
| factor(X25.CS2)=0 | 4.6 | 351 | 0 | 4 | 0.5410 | 0.5113 | 0.5723 |
| factor(X25.CS2)=0 | 4.61 | 347 | 0 | 3 | 0.5410 | 0.5113 | 0.5723 |
| factor(X25.CS2)=0 | 4.62 | 344 | 0 | 1 | 0.5410 | 0.5113 | 0.5723 |
| factor(X25.CS2)=0 | 4.64 | 343 | 0 | 1 | 0.5410 | 0.5113 | 0.5723 |
| factor(X25.CS2)=0 | 4.65 | 342 | 0 | 1 | 0.5410 | 0.5113 | 0.5723 |
| factor(X25.CS2)=0 | 4.68 | 341 | 0 | 1 | 0.5410 | 0.5113 | 0.5723 |
| factor(X25.CS2)=0 | 4.7 | 340 | 0 | 1 | 0.5410 | 0.5113 | 0.5723 |
| factor(X25.CS2)=0 | 4.72 | 339 | 0 | 2 | 0.5410 | 0.5113 | 0.5723 |
| factor(X25.CS2)=0 | 4.73 | 337 | 0 | 2 | 0.5410 | 0.5113 | 0.5723 |
| factor(X25.CS2)=0 | 4.75 | 335 | 0 | 1 | 0.5410 | 0.5113 | 0.5723 |
| factor(X25.CS2)=0 | 4.79 | 334 | 0 | 1 | 0.5410 | 0.5113 | 0.5723 |
| factor(X25.CS2)=0 | 4.81 | 333 | 0 | 2 | 0.5410 | 0.5113 | 0.5723 |
| factor(X25.CS2)=0 | 4.82 | 331 | 0 | 1 | 0.5410 | 0.5113 | 0.5723 |
| factor(X25.CS2)=0 | 4.85 | 330 | 0 | 4 | 0.5410 | 0.5113 | 0.5723 |
| factor(X25.CS2)=0 | 4.88 | 326 | 0 | 1 | 0.5410 | 0.5113 | 0.5723 |
| factor(X25.CS2)=0 | 4.89 | 325 | 0 | 1 | 0.5410 | 0.5113 | 0.5723 |
| factor(X25.CS2)=0 | 4.9 | 324 | 0 | 1 | 0.5410 | 0.5113 | 0.5723 |
| factor(X25.CS2)=0 | 4.91 | 323 | 0 | 1 | 0.5410 | 0.5113 | 0.5723 |
| factor(X25.CS2)=0 | 4.94 | 322 | 0 | 2 | 0.5410 | 0.5113 | 0.5723 |
| factor(X25.CS2)=0 | 4.97 | 320 | 0 | 1 | 0.5410 | 0.5113 | 0.5723 |
| factor(X25.CS2)=0 | 4.99 | 319 | 0 | 1 | 0.5410 | 0.5113 | 0.5723 |
| factor(X25.CS2)=0 | 5 | 318 | 0 | 1 | 0.5410 | 0.5113 | 0.5723 |
| factor(X25.CS2)=0 | 5.02 | 317 | 0 | 1 | 0.5410 | 0.5113 | 0.5723 |
| factor(X25.CS2)=0 | 5.03 | 316 | 0 | 1 | 0.5410 | 0.5113 | 0.5723 |
| factor(X25.CS2)=0 | 5.04 | 315 | 0 | 1 | 0.5410 | 0.5113 | 0.5723 |
| factor(X25.CS2)=0 | 5.05 | 314 | 0 | 2 | 0.5410 | 0.5113 | 0.5723 |
| factor(X25.CS2)=0 | 5.08 | 312 | 0 | 1 | 0.5410 | 0.5113 | 0.5723 |
| factor(X25.CS2)=0 | 5.1 | 311 | 0 | 1 | 0.5410 | 0.5113 | 0.5723 |
| factor(X25.CS2)=0 | 5.12 | 310 | 1 | 1 | 0.5392 | 0.5095 | 0.5707 |
| factor(X25.CS2)=0 | 5.14 | 308 | 0 | 1 | 0.5392 | 0.5095 | 0.5707 |
| factor(X25.CS2)=0 | 5.15 | 307 | 0 | 2 | 0.5392 | 0.5095 | 0.5707 |
| factor(X25.CS2)=0 | 5.16 | 305 | 0 | 1 | 0.5392 | 0.5095 | 0.5707 |
| factor(X25.CS2)=0 | 5.17 | 304 | 0 | 1 | 0.5392 | 0.5095 | 0.5707 |
| factor(X25.CS2)=0 | 5.18 | 303 | 0 | 3 | 0.5392 | 0.5095 | 0.5707 |
| factor(X25.CS2)=0 | 5.2 | 300 | 0 | 2 | 0.5392 | 0.5095 | 0.5707 |
| factor(X25.CS2)=0 | 5.22 | 298 | 0 | 1 | 0.5392 | 0.5095 | 0.5707 |
| factor(X25.CS2)=0 | 5.25 | 297 | 0 | 2 | 0.5392 | 0.5095 | 0.5707 |
| factor(X25.CS2)=0 | 5.26 | 295 | 0 | 2 | 0.5392 | 0.5095 | 0.5707 |
| factor(X25.CS2)=0 | 5.27 | 293 | 0 | 2 | 0.5392 | 0.5095 | 0.5707 |
| factor(X25.CS2)=0 | 5.29 | 291 | 0 | 2 | 0.5392 | 0.5095 | 0.5707 |
| factor(X25.CS2)=0 | 5.3 | 289 | 0 | 1 | 0.5392 | 0.5095 | 0.5707 |
| factor(X25.CS2)=0 | 5.31 | 288 | 0 | 1 | 0.5392 | 0.5095 | 0.5707 |
| factor(X25.CS2)=0 | 5.32 | 287 | 0 | 2 | 0.5392 | 0.5095 | 0.5707 |
| factor(X25.CS2)=0 | 5.33 | 285 | 0 | 2 | 0.5392 | 0.5095 | 0.5707 |
| factor(X25.CS2)=0 | 5.34 | 283 | 0 | 2 | 0.5392 | 0.5095 | 0.5707 |
| factor(X25.CS2)=0 | 5.35 | 281 | 0 | 6 | 0.5392 | 0.5095 | 0.5707 |
| factor(X25.CS2)=0 | 5.36 | 275 | 0 | 2 | 0.5392 | 0.5095 | 0.5707 |
| factor(X25.CS2)=0 | 5.37 | 273 | 0 | 2 | 0.5392 | 0.5095 | 0.5707 |
| factor(X25.CS2)=0 | 5.38 | 271 | 0 | 2 | 0.5392 | 0.5095 | 0.5707 |
| factor(X25.CS2)=0 | 5.39 | 269 | 0 | 1 | 0.5392 | 0.5095 | 0.5707 |
| factor(X25.CS2)=0 | 5.4 | 268 | 0 | 1 | 0.5392 | 0.5095 | 0.5707 |
| factor(X25.CS2)=0 | 5.41 | 267 | 0 | 2 | 0.5392 | 0.5095 | 0.5707 |
| factor(X25.CS2)=0 | 5.42 | 265 | 0 | 4 | 0.5392 | 0.5095 | 0.5707 |
| factor(X25.CS2)=0 | 5.43 | 261 | 0 | 1 | 0.5392 | 0.5095 | 0.5707 |
| factor(X25.CS2)=0 | 5.44 | 260 | 0 | 4 | 0.5392 | 0.5095 | 0.5707 |
| factor(X25.CS2)=0 | 5.45 | 256 | 0 | 1 | 0.5392 | 0.5095 | 0.5707 |
| factor(X25.CS2)=0 | 5.46 | 255 | 0 | 2 | 0.5392 | 0.5095 | 0.5707 |
| factor(X25.CS2)=0 | 5.48 | 253 | 0 | 3 | 0.5392 | 0.5095 | 0.5707 |
| factor(X25.CS2)=0 | 5.49 | 250 | 0 | 1 | 0.5392 | 0.5095 | 0.5707 |
| factor(X25.CS2)=0 | 5.5 | 249 | 0 | 1 | 0.5392 | 0.5095 | 0.5707 |
| factor(X25.CS2)=0 | 5.51 | 248 | 0 | 2 | 0.5392 | 0.5095 | 0.5707 |
| factor(X25.CS2)=0 | 5.52 | 246 | 0 | 2 | 0.5392 | 0.5095 | 0.5707 |
| factor(X25.CS2)=0 | 5.54 | 244 | 0 | 4 | 0.5392 | 0.5095 | 0.5707 |
| factor(X25.CS2)=0 | 5.55 | 240 | 0 | 2 | 0.5392 | 0.5095 | 0.5707 |
| factor(X25.CS2)=0 | 5.56 | 238 | 0 | 2 | 0.5392 | 0.5095 | 0.5707 |
| factor(X25.CS2)=0 | 5.57 | 236 | 0 | 1 | 0.5392 | 0.5095 | 0.5707 |
| factor(X25.CS2)=0 | 5.58 | 235 | 1 | 1 | 0.5369 | 0.5070 | 0.5686 |
| factor(X25.CS2)=0 | 5.6 | 233 | 0 | 1 | 0.5369 | 0.5070 | 0.5686 |
| factor(X25.CS2)=0 | 5.61 | 232 | 0 | 1 | 0.5369 | 0.5070 | 0.5686 |
| factor(X25.CS2)=0 | 5.63 | 231 | 0 | 2 | 0.5369 | 0.5070 | 0.5686 |
| factor(X25.CS2)=0 | 5.65 | 229 | 0 | 1 | 0.5369 | 0.5070 | 0.5686 |
| factor(X25.CS2)=0 | 5.66 | 228 | 0 | 1 | 0.5369 | 0.5070 | 0.5686 |
| factor(X25.CS2)=0 | 5.67 | 227 | 0 | 3 | 0.5369 | 0.5070 | 0.5686 |
| factor(X25.CS2)=0 | 5.7 | 224 | 0 | 3 | 0.5369 | 0.5070 | 0.5686 |
| factor(X25.CS2)=0 | 5.71 | 221 | 0 | 2 | 0.5369 | 0.5070 | 0.5686 |
| factor(X25.CS2)=0 | 5.72 | 219 | 0 | 1 | 0.5369 | 0.5070 | 0.5686 |
| factor(X25.CS2)=0 | 5.73 | 218 | 0 | 2 | 0.5369 | 0.5070 | 0.5686 |
| factor(X25.CS2)=0 | 5.74 | 216 | 0 | 1 | 0.5369 | 0.5070 | 0.5686 |
| factor(X25.CS2)=0 | 5.75 | 215 | 0 | 2 | 0.5369 | 0.5070 | 0.5686 |
| factor(X25.CS2)=0 | 5.77 | 213 | 0 | 1 | 0.5369 | 0.5070 | 0.5686 |
| factor(X25.CS2)=0 | 5.78 | 212 | 0 | 2 | 0.5369 | 0.5070 | 0.5686 |
| factor(X25.CS2)=0 | 5.79 | 210 | 0 | 1 | 0.5369 | 0.5070 | 0.5686 |
| factor(X25.CS2)=0 | 5.8 | 209 | 0 | 1 | 0.5369 | 0.5070 | 0.5686 |
| factor(X25.CS2)=0 | 5.81 | 208 | 0 | 2 | 0.5369 | 0.5070 | 0.5686 |
| factor(X25.CS2)=0 | 5.84 | 206 | 0 | 2 | 0.5369 | 0.5070 | 0.5686 |
| factor(X25.CS2)=0 | 5.85 | 204 | 0 | 1 | 0.5369 | 0.5070 | 0.5686 |
| factor(X25.CS2)=0 | 5.88 | 203 | 0 | 1 | 0.5369 | 0.5070 | 0.5686 |
| factor(X25.CS2)=0 | 5.89 | 202 | 0 | 2 | 0.5369 | 0.5070 | 0.5686 |
| factor(X25.CS2)=0 | 5.92 | 200 | 0 | 1 | 0.5369 | 0.5070 | 0.5686 |
| factor(X25.CS2)=0 | 5.93 | 199 | 0 | 1 | 0.5369 | 0.5070 | 0.5686 |
| factor(X25.CS2)=0 | 5.94 | 198 | 0 | 2 | 0.5369 | 0.5070 | 0.5686 |
| factor(X25.CS2)=0 | 5.95 | 196 | 0 | 3 | 0.5369 | 0.5070 | 0.5686 |
| factor(X25.CS2)=0 | 5.96 | 193 | 0 | 1 | 0.5369 | 0.5070 | 0.5686 |
| factor(X25.CS2)=0 | 5.98 | 192 | 0 | 1 | 0.5369 | 0.5070 | 0.5686 |
| factor(X25.CS2)=0 | 6 | 191 | 0 | 6 | 0.5369 | 0.5070 | 0.5686 |
| factor(X25.CS2)=0 | 6.01 | 185 | 0 | 1 | 0.5369 | 0.5070 | 0.5686 |
| factor(X25.CS2)=0 | 6.02 | 184 | 0 | 1 | 0.5369 | 0.5070 | 0.5686 |
| factor(X25.CS2)=0 | 6.04 | 183 | 0 | 2 | 0.5369 | 0.5070 | 0.5686 |
| factor(X25.CS2)=0 | 6.07 | 181 | 0 | 1 | 0.5369 | 0.5070 | 0.5686 |
| factor(X25.CS2)=0 | 6.08 | 180 | 0 | 1 | 0.5369 | 0.5070 | 0.5686 |
| factor(X25.CS2)=0 | 6.12 | 179 | 0 | 2 | 0.5369 | 0.5070 | 0.5686 |
| factor(X25.CS2)=0 | 6.15 | 177 | 0 | 3 | 0.5369 | 0.5070 | 0.5686 |
| factor(X25.CS2)=0 | 6.16 | 174 | 0 | 2 | 0.5369 | 0.5070 | 0.5686 |
| factor(X25.CS2)=0 | 6.17 | 172 | 0 | 1 | 0.5369 | 0.5070 | 0.5686 |
| factor(X25.CS2)=0 | 6.18 | 171 | 0 | 2 | 0.5369 | 0.5070 | 0.5686 |
| factor(X25.CS2)=0 | 6.22 | 169 | 0 | 1 | 0.5369 | 0.5070 | 0.5686 |
| factor(X25.CS2)=0 | 6.25 | 168 | 0 | 2 | 0.5369 | 0.5070 | 0.5686 |
| factor(X25.CS2)=0 | 6.27 | 166 | 0 | 1 | 0.5369 | 0.5070 | 0.5686 |
| factor(X25.CS2)=0 | 6.28 | 165 | 0 | 1 | 0.5369 | 0.5070 | 0.5686 |
| factor(X25.CS2)=0 | 6.29 | 164 | 0 | 1 | 0.5369 | 0.5070 | 0.5686 |
| factor(X25.CS2)=0 | 6.32 | 163 | 0 | 1 | 0.5369 | 0.5070 | 0.5686 |
| factor(X25.CS2)=0 | 6.35 | 162 | 0 | 2 | 0.5369 | 0.5070 | 0.5686 |
| factor(X25.CS2)=0 | 6.36 | 160 | 0 | 1 | 0.5369 | 0.5070 | 0.5686 |
| factor(X25.CS2)=0 | 6.37 | 159 | 0 | 2 | 0.5369 | 0.5070 | 0.5686 |
| factor(X25.CS2)=0 | 6.38 | 157 | 0 | 2 | 0.5369 | 0.5070 | 0.5686 |
| factor(X25.CS2)=0 | 6.39 | 155 | 0 | 4 | 0.5369 | 0.5070 | 0.5686 |
| factor(X25.CS2)=0 | 6.4 | 151 | 0 | 2 | 0.5369 | 0.5070 | 0.5686 |
| factor(X25.CS2)=0 | 6.42 | 149 | 0 | 3 | 0.5369 | 0.5070 | 0.5686 |
| factor(X25.CS2)=0 | 6.45 | 146 | 3 | 1 | 0.5259 | 0.4943 | 0.5595 |
| factor(X25.CS2)=0 | 6.46 | 142 | 0 | 2 | 0.5259 | 0.4943 | 0.5595 |
| factor(X25.CS2)=0 | 6.47 | 140 | 0 | 3 | 0.5259 | 0.4943 | 0.5595 |
| factor(X25.CS2)=0 | 6.48 | 137 | 0 | 2 | 0.5259 | 0.4943 | 0.5595 |
| factor(X25.CS2)=0 | 6.49 | 135 | 0 | 1 | 0.5259 | 0.4943 | 0.5595 |
| factor(X25.CS2)=0 | 6.5 | 134 | 0 | 2 | 0.5259 | 0.4943 | 0.5595 |
| factor(X25.CS2)=0 | 6.51 | 132 | 0 | 2 | 0.5259 | 0.4943 | 0.5595 |
| factor(X25.CS2)=0 | 6.52 | 130 | 0 | 1 | 0.5259 | 0.4943 | 0.5595 |
| factor(X25.CS2)=0 | 6.53 | 129 | 0 | 1 | 0.5259 | 0.4943 | 0.5595 |
| factor(X25.CS2)=0 | 6.54 | 128 | 0 | 1 | 0.5259 | 0.4943 | 0.5595 |
| factor(X25.CS2)=0 | 6.55 | 127 | 0 | 1 | 0.5259 | 0.4943 | 0.5595 |
| factor(X25.CS2)=0 | 6.56 | 126 | 0 | 2 | 0.5259 | 0.4943 | 0.5595 |
| factor(X25.CS2)=0 | 6.57 | 124 | 0 | 2 | 0.5259 | 0.4943 | 0.5595 |
| factor(X25.CS2)=0 | 6.58 | 122 | 0 | 2 | 0.5259 | 0.4943 | 0.5595 |
| factor(X25.CS2)=0 | 6.59 | 120 | 0 | 1 | 0.5259 | 0.4943 | 0.5595 |
| factor(X25.CS2)=0 | 6.6 | 119 | 0 | 1 | 0.5259 | 0.4943 | 0.5595 |
| factor(X25.CS2)=0 | 6.63 | 118 | 0 | 1 | 0.5259 | 0.4943 | 0.5595 |
| factor(X25.CS2)=0 | 6.64 | 117 | 0 | 2 | 0.5259 | 0.4943 | 0.5595 |
| factor(X25.CS2)=0 | 6.65 | 115 | 0 | 3 | 0.5259 | 0.4943 | 0.5595 |
| factor(X25.CS2)=0 | 6.68 | 112 | 0 | 1 | 0.5259 | 0.4943 | 0.5595 |
| factor(X25.CS2)=0 | 6.69 | 111 | 0 | 1 | 0.5259 | 0.4943 | 0.5595 |
| factor(X25.CS2)=0 | 6.73 | 110 | 0 | 1 | 0.5259 | 0.4943 | 0.5595 |
| factor(X25.CS2)=0 | 6.75 | 109 | 0 | 3 | 0.5259 | 0.4943 | 0.5595 |
| factor(X25.CS2)=0 | 6.77 | 106 | 0 | 1 | 0.5259 | 0.4943 | 0.5595 |
| factor(X25.CS2)=0 | 6.78 | 105 | 0 | 1 | 0.5259 | 0.4943 | 0.5595 |
| factor(X25.CS2)=0 | 6.79 | 104 | 0 | 2 | 0.5259 | 0.4943 | 0.5595 |
| factor(X25.CS2)=0 | 6.81 | 102 | 0 | 2 | 0.5259 | 0.4943 | 0.5595 |
| factor(X25.CS2)=0 | 6.85 | 100 | 0 | 1 | 0.5259 | 0.4943 | 0.5595 |
| factor(X25.CS2)=0 | 6.87 | 99 | 0 | 1 | 0.5259 | 0.4943 | 0.5595 |
| factor(X25.CS2)=0 | 6.89 | 98 | 0 | 2 | 0.5259 | 0.4943 | 0.5595 |
| factor(X25.CS2)=0 | 6.91 | 96 | 0 | 1 | 0.5259 | 0.4943 | 0.5595 |
| factor(X25.CS2)=0 | 6.92 | 95 | 0 | 1 | 0.5259 | 0.4943 | 0.5595 |
| factor(X25.CS2)=0 | 6.94 | 94 | 0 | 1 | 0.5259 | 0.4943 | 0.5595 |
| factor(X25.CS2)=0 | 6.96 | 93 | 0 | 4 | 0.5259 | 0.4943 | 0.5595 |
| factor(X25.CS2)=0 | 6.98 | 89 | 0 | 1 | 0.5259 | 0.4943 | 0.5595 |
| factor(X25.CS2)=0 | 7 | 88 | 0 | 1 | 0.5259 | 0.4943 | 0.5595 |
| factor(X25.CS2)=0 | 7.08 | 87 | 0 | 1 | 0.5259 | 0.4943 | 0.5595 |
| factor(X25.CS2)=0 | 7.14 | 86 | 0 | 1 | 0.5259 | 0.4943 | 0.5595 |
| factor(X25.CS2)=0 | 7.16 | 85 | 0 | 1 | 0.5259 | 0.4943 | 0.5595 |
| factor(X25.CS2)=0 | 7.2 | 84 | 0 | 1 | 0.5259 | 0.4943 | 0.5595 |
| factor(X25.CS2)=0 | 7.21 | 83 | 0 | 1 | 0.5259 | 0.4943 | 0.5595 |
| factor(X25.CS2)=0 | 7.26 | 82 | 0 | 3 | 0.5259 | 0.4943 | 0.5595 |
| factor(X25.CS2)=0 | 7.29 | 79 | 0 | 1 | 0.5259 | 0.4943 | 0.5595 |
| factor(X25.CS2)=0 | 7.35 | 78 | 0 | 2 | 0.5259 | 0.4943 | 0.5595 |
| factor(X25.CS2)=0 | 7.36 | 76 | 0 | 6 | 0.5259 | 0.4943 | 0.5595 |
| factor(X25.CS2)=0 | 7.37 | 70 | 0 | 1 | 0.5259 | 0.4943 | 0.5595 |
| factor(X25.CS2)=0 | 7.38 | 69 | 0 | 1 | 0.5259 | 0.4943 | 0.5595 |
| factor(X25.CS2)=0 | 7.39 | 68 | 0 | 2 | 0.5259 | 0.4943 | 0.5595 |
| factor(X25.CS2)=0 | 7.4 | 66 | 0 | 1 | 0.5259 | 0.4943 | 0.5595 |
| factor(X25.CS2)=0 | 7.41 | 65 | 0 | 1 | 0.5259 | 0.4943 | 0.5595 |
| factor(X25.CS2)=0 | 7.42 | 64 | 0 | 1 | 0.5259 | 0.4943 | 0.5595 |
| factor(X25.CS2)=0 | 7.45 | 63 | 0 | 1 | 0.5259 | 0.4943 | 0.5595 |
| factor(X25.CS2)=0 | 7.47 | 62 | 0 | 1 | 0.5259 | 0.4943 | 0.5595 |
| factor(X25.CS2)=0 | 7.48 | 61 | 0 | 2 | 0.5259 | 0.4943 | 0.5595 |
| factor(X25.CS2)=0 | 7.5 | 59 | 0 | 1 | 0.5259 | 0.4943 | 0.5595 |
| factor(X25.CS2)=0 | 7.53 | 58 | 0 | 1 | 0.5259 | 0.4943 | 0.5595 |
| factor(X25.CS2)=0 | 7.54 | 57 | 0 | 1 | 0.5259 | 0.4943 | 0.5595 |
| factor(X25.CS2)=0 | 7.55 | 56 | 0 | 1 | 0.5259 | 0.4943 | 0.5595 |
| factor(X25.CS2)=0 | 7.56 | 55 | 0 | 2 | 0.5259 | 0.4943 | 0.5595 |
| factor(X25.CS2)=0 | 7.58 | 53 | 0 | 4 | 0.5259 | 0.4943 | 0.5595 |
| factor(X25.CS2)=0 | 7.59 | 49 | 0 | 1 | 0.5259 | 0.4943 | 0.5595 |
| factor(X25.CS2)=0 | 7.6 | 48 | 0 | 2 | 0.5259 | 0.4943 | 0.5595 |
| factor(X25.CS2)=0 | 7.61 | 46 | 0 | 1 | 0.5259 | 0.4943 | 0.5595 |
| factor(X25.CS2)=0 | 7.62 | 45 | 0 | 1 | 0.5259 | 0.4943 | 0.5595 |
| factor(X25.CS2)=0 | 7.67 | 44 | 0 | 1 | 0.5259 | 0.4943 | 0.5595 |
| factor(X25.CS2)=0 | 7.68 | 43 | 0 | 1 | 0.5259 | 0.4943 | 0.5595 |
| factor(X25.CS2)=0 | 7.69 | 42 | 0 | 2 | 0.5259 | 0.4943 | 0.5595 |
| factor(X25.CS2)=0 | 7.73 | 40 | 0 | 2 | 0.5259 | 0.4943 | 0.5595 |
| factor(X25.CS2)=0 | 7.74 | 38 | 0 | 3 | 0.5259 | 0.4943 | 0.5595 |
| factor(X25.CS2)=0 | 7.81 | 35 | 0 | 1 | 0.5259 | 0.4943 | 0.5595 |
| factor(X25.CS2)=0 | 7.82 | 34 | 0 | 1 | 0.5259 | 0.4943 | 0.5595 |
| factor(X25.CS2)=0 | 7.9 | 33 | 0 | 1 | 0.5259 | 0.4943 | 0.5595 |
| factor(X25.CS2)=0 | 7.94 | 32 | 0 | 2 | 0.5259 | 0.4943 | 0.5595 |
| factor(X25.CS2)=0 | 7.98 | 30 | 0 | 1 | 0.5259 | 0.4943 | 0.5595 |
| factor(X25.CS2)=0 | 7.99 | 29 | 1 | 1 | 0.5078 | 0.4629 | 0.5570 |
| factor(X25.CS2)=0 | 8.01 | 27 | 0 | 1 | 0.5078 | 0.4629 | 0.5570 |
| factor(X25.CS2)=0 | 8.06 | 26 | 0 | 1 | 0.5078 | 0.4629 | 0.5570 |
| factor(X25.CS2)=0 | 8.07 | 25 | 0 | 1 | 0.5078 | 0.4629 | 0.5570 |
| factor(X25.CS2)=0 | 8.17 | 24 | 0 | 1 | 0.5078 | 0.4629 | 0.5570 |
| factor(X25.CS2)=0 | 8.21 | 23 | 0 | 1 | 0.5078 | 0.4629 | 0.5570 |
| factor(X25.CS2)=0 | 8.26 | 22 | 0 | 1 | 0.5078 | 0.4629 | 0.5570 |
| factor(X25.CS2)=0 | 8.27 | 21 | 0 | 1 | 0.5078 | 0.4629 | 0.5570 |
| factor(X25.CS2)=0 | 8.28 | 20 | 0 | 1 | 0.5078 | 0.4629 | 0.5570 |
| factor(X25.CS2)=0 | 8.29 | 19 | 0 | 1 | 0.5078 | 0.4629 | 0.5570 |
| factor(X25.CS2)=0 | 8.36 | 18 | 0 | 1 | 0.5078 | 0.4629 | 0.5570 |
| factor(X25.CS2)=0 | 8.48 | 17 | 0 | 1 | 0.5078 | 0.4629 | 0.5570 |
| factor(X25.CS2)=0 | 8.49 | 16 | 0 | 2 | 0.5078 | 0.4629 | 0.5570 |
| factor(X25.CS2)=0 | 8.5 | 14 | 0 | 1 | 0.5078 | 0.4629 | 0.5570 |
| factor(X25.CS2)=0 | 8.53 | 13 | 0 | 1 | 0.5078 | 0.4629 | 0.5570 |
| factor(X25.CS2)=0 | 8.72 | 12 | 0 | 1 | 0.5078 | 0.4629 | 0.5570 |
| factor(X25.CS2)=0 | 8.84 | 11 | 0 | 1 | 0.5078 | 0.4629 | 0.5570 |
| factor(X25.CS2)=0 | 8.88 | 10 | 0 | 3 | 0.5078 | 0.4629 | 0.5570 |
| factor(X25.CS2)=0 | 9.04 | 7 | 0 | 1 | 0.5078 | 0.4629 | 0.5570 |
| factor(X25.CS2)=0 | 9.06 | 6 | 0 | 2 | 0.5078 | 0.4629 | 0.5570 |
| factor(X25.CS2)=0 | 9.08 | 4 | 0 | 1 | 0.5078 | 0.4629 | 0.5570 |
| factor(X25.CS2)=0 | 9.26 | 3 | 0 | 1 | 0.5078 | 0.4629 | 0.5570 |
| factor(X25.CS2)=0 | 9.61 | 2 | 0 | 1 | 0.5078 | 0.4629 | 0.5570 |
| factor(X25.CS2)=0 | 9.95 | 1 | 0 | 1 | 0.5078 | 0.4629 | 0.5570 |
| factor(X25.CS2)=1 | 0.21 | 16 | 1 | 0 | 0.9375 | 0.8261 | 1.0000 |
| factor(X25.CS2)=1 | 0.35 | 15 | 1 | 0 | 0.8750 | 0.7271 | 1.0000 |
| factor(X25.CS2)=1 | 0.53 | 14 | 1 | 0 | 0.8125 | 0.6421 | 1.0000 |
| factor(X25.CS2)=1 | 0.55 | 13 | 1 | 0 | 0.7500 | 0.5652 | 0.9952 |
| factor(X25.CS2)=1 | 0.7 | 12 | 2 | 0 | 0.6250 | 0.4276 | 0.9135 |
| factor(X25.CS2)=1 | 0.75 | 10 | 1 | 0 | 0.5625 | 0.3651 | 0.8666 |
| factor(X25.CS2)=1 | 0.8 | 9 | 1 | 0 | 0.5000 | 0.3063 | 0.8162 |
| factor(X25.CS2)=1 | 0.86 | 8 | 1 | 0 | 0.4375 | 0.2510 | 0.7626 |
| factor(X25.CS2)=1 | 0.9 | 7 | 1 | 0 | 0.3750 | 0.1992 | 0.7059 |
| factor(X25.CS2)=1 | 1.12 | 6 | 1 | 0 | 0.3125 | 0.1511 | 0.6464 |
| factor(X25.CS2)=1 | 1.98 | 5 | 2 | 0 | 0.1875 | 0.0676 | 0.5200 |
| factor(X25.CS2)=1 | 2.13 | 3 | 1 | 0 | 0.1250 | 0.0342 | 0.4570 |
| factor(X25.CS2)=1 | 5.81 | 2 | 0 | 1 | 0.1250 | 0.0342 | 0.4570 |
| factor(X25.CS2)=1 | 7.48 | 1 | 0 | 1 | 0.1250 | 0.0342 | 0.4570 |
| factor(X25.CS2)=2 | 0.13 | 132 | 2 | 0 | 0.9848 | 0.9642 | 1.0000 |
| factor(X25.CS2)=2 | 0.15 | 130 | 2 | 0 | 0.9697 | 0.9409 | 0.9994 |
| factor(X25.CS2)=2 | 0.16 | 128 | 5 | 0 | 0.9318 | 0.8898 | 0.9758 |
| factor(X25.CS2)=2 | 0.17 | 123 | 4 | 0 | 0.9015 | 0.8521 | 0.9538 |
| factor(X25.CS2)=2 | 0.18 | 119 | 2 | 0 | 0.8864 | 0.8338 | 0.9422 |
| factor(X25.CS2)=2 | 0.19 | 117 | 10 | 0 | 0.8106 | 0.7464 | 0.8803 |
| factor(X25.CS2)=2 | 0.2 | 107 | 3 | 0 | 0.7879 | 0.7211 | 0.8608 |
| factor(X25.CS2)=2 | 0.21 | 104 | 10 | 0 | 0.7121 | 0.6389 | 0.7937 |
| factor(X25.CS2)=2 | 0.22 | 94 | 9 | 0 | 0.6439 | 0.5672 | 0.7310 |
| factor(X25.CS2)=2 | 0.23 | 85 | 8 | 0 | 0.5833 | 0.5050 | 0.6738 |
| factor(X25.CS2)=2 | 0.24 | 77 | 3 | 0 | 0.5606 | 0.4820 | 0.6520 |
| factor(X25.CS2)=2 | 0.25 | 74 | 2 | 0 | 0.5455 | 0.4668 | 0.6374 |
| factor(X25.CS2)=2 | 0.27 | 72 | 1 | 0 | 0.5379 | 0.4592 | 0.6300 |
| factor(X25.CS2)=2 | 0.28 | 71 | 3 | 0 | 0.5152 | 0.4366 | 0.6079 |
| factor(X25.CS2)=2 | 0.29 | 68 | 1 | 0 | 0.5076 | 0.4291 | 0.6004 |
| factor(X25.CS2)=2 | 0.3 | 67 | 3 | 0 | 0.4848 | 0.4067 | 0.5781 |
| factor(X25.CS2)=2 | 0.32 | 64 | 1 | 0 | 0.4773 | 0.3992 | 0.5706 |
| factor(X25.CS2)=2 | 0.34 | 63 | 1 | 0 | 0.4697 | 0.3918 | 0.5630 |
| factor(X25.CS2)=2 | 0.47 | 62 | 2 | 0 | 0.4545 | 0.3771 | 0.5479 |
| factor(X25.CS2)=2 | 0.49 | 60 | 3 | 0 | 0.4318 | 0.3551 | 0.5252 |
| factor(X25.CS2)=2 | 0.5 | 57 | 1 | 0 | 0.4242 | 0.3478 | 0.5175 |
| factor(X25.CS2)=2 | 0.53 | 56 | 1 | 0 | 0.4167 | 0.3405 | 0.5099 |
| factor(X25.CS2)=2 | 0.59 | 55 | 1 | 0 | 0.4091 | 0.3333 | 0.5022 |
| factor(X25.CS2)=2 | 0.62 | 54 | 1 | 0 | 0.4015 | 0.3260 | 0.4945 |
| factor(X25.CS2)=2 | 0.64 | 53 | 3 | 0 | 0.3788 | 0.3045 | 0.4713 |
| factor(X25.CS2)=2 | 0.69 | 50 | 1 | 0 | 0.3712 | 0.2973 | 0.4635 |
| factor(X25.CS2)=2 | 0.72 | 49 | 1 | 0 | 0.3636 | 0.2902 | 0.4557 |
| factor(X25.CS2)=2 | 0.74 | 48 | 2 | 0 | 0.3485 | 0.2760 | 0.4400 |
| factor(X25.CS2)=2 | 0.77 | 46 | 2 | 0 | 0.3333 | 0.2619 | 0.4243 |
| factor(X25.CS2)=2 | 0.78 | 44 | 1 | 0 | 0.3258 | 0.2549 | 0.4164 |
| factor(X25.CS2)=2 | 0.86 | 43 | 0 | 1 | 0.3258 | 0.2549 | 0.4164 |
| factor(X25.CS2)=2 | 0.87 | 42 | 1 | 0 | 0.3180 | 0.2477 | 0.4083 |
| factor(X25.CS2)=2 | 0.91 | 41 | 1 | 0 | 0.3102 | 0.2405 | 0.4002 |
| factor(X25.CS2)=2 | 0.95 | 40 | 1 | 0 | 0.3025 | 0.2334 | 0.3921 |
| factor(X25.CS2)=2 | 1 | 39 | 1 | 0 | 0.2947 | 0.2263 | 0.3839 |
| factor(X25.CS2)=2 | 1.02 | 38 | 1 | 0 | 0.2870 | 0.2192 | 0.3757 |
| factor(X25.CS2)=2 | 1.07 | 37 | 1 | 0 | 0.2792 | 0.2121 | 0.3675 |
| factor(X25.CS2)=2 | 1.15 | 36 | 2 | 0 | 0.2637 | 0.1981 | 0.3510 |
| factor(X25.CS2)=2 | 1.17 | 34 | 1 | 0 | 0.2560 | 0.1912 | 0.3427 |
| factor(X25.CS2)=2 | 1.38 | 33 | 1 | 0 | 0.2482 | 0.1842 | 0.3344 |
| factor(X25.CS2)=2 | 1.42 | 32 | 3 | 0 | 0.2249 | 0.1636 | 0.3092 |
| factor(X25.CS2)=2 | 1.74 | 29 | 1 | 0 | 0.2172 | 0.1568 | 0.3007 |
| factor(X25.CS2)=2 | 2.26 | 28 | 1 | 0 | 0.2094 | 0.1501 | 0.2922 |
| factor(X25.CS2)=2 | 2.72 | 27 | 3 | 0 | 0.1861 | 0.1300 | 0.2665 |
| factor(X25.CS2)=2 | 3.39 | 24 | 2 | 0 | 0.1706 | 0.1168 | 0.2492 |
| factor(X25.CS2)=2 | 3.68 | 22 | 0 | 1 | 0.1706 | 0.1168 | 0.2492 |
| factor(X25.CS2)=2 | 4.61 | 21 | 0 | 1 | 0.1706 | 0.1168 | 0.2492 |
| factor(X25.CS2)=2 | 4.87 | 20 | 0 | 2 | 0.1706 | 0.1168 | 0.2492 |
| factor(X25.CS2)=2 | 5.33 | 18 | 0 | 1 | 0.1706 | 0.1168 | 0.2492 |
| factor(X25.CS2)=2 | 5.38 | 17 | 0 | 1 | 0.1706 | 0.1168 | 0.2492 |
| factor(X25.CS2)=2 | 5.4 | 16 | 0 | 1 | 0.1706 | 0.1168 | 0.2492 |
| factor(X25.CS2)=2 | 6.19 | 15 | 0 | 1 | 0.1706 | 0.1168 | 0.2492 |
| factor(X25.CS2)=2 | 6.2 | 14 | 0 | 1 | 0.1706 | 0.1168 | 0.2492 |
| factor(X25.CS2)=2 | 6.4 | 13 | 0 | 1 | 0.1706 | 0.1168 | 0.2492 |
| factor(X25.CS2)=2 | 6.51 | 12 | 0 | 1 | 0.1706 | 0.1168 | 0.2492 |
| factor(X25.CS2)=2 | 7 | 11 | 0 | 2 | 0.1706 | 0.1168 | 0.2492 |
| factor(X25.CS2)=2 | 7.12 | 9 | 0 | 1 | 0.1706 | 0.1168 | 0.2492 |
| factor(X25.CS2)=2 | 7.27 | 8 | 0 | 1 | 0.1706 | 0.1168 | 0.2492 |
| factor(X25.CS2)=2 | 7.46 | 7 | 0 | 1 | 0.1706 | 0.1168 | 0.2492 |
| factor(X25.CS2)=2 | 7.72 | 6 | 0 | 1 | 0.1706 | 0.1168 | 0.2492 |
| factor(X25.CS2)=2 | 7.74 | 5 | 0 | 2 | 0.1706 | 0.1168 | 0.2492 |
| factor(X25.CS2)=2 | 8.1 | 3 | 0 | 1 | 0.1706 | 0.1168 | 0.2492 |
| factor(X25.CS2)=2 | 8.15 | 2 | 0 | 1 | 0.1706 | 0.1168 | 0.2492 |
| factor(X25.CS2)=2 | 8.69 | 1 | 0 | 1 | 0.1706 | 0.1168 | 0.2492 |

|  |  |  |  |  |  |  |  |  |  |
| --- | --- | --- | --- | --- | --- | --- | --- | --- | --- |
| X25.CS2 | records | n.max | n.start | events | \*rmean | \*se(rmean) | median | 0.95LCL | 0.95UCL |
| factor(X25.CS2)=0 | 1057 | 1057 | 1057 | 483 | 5.12 | 0.12 | NA | 6.45 | NA |
| factor(X25.CS2)=1 | 16 | 16 | 16 | 14 | 1.934 | 0.653 | 0.83 | 0.7 | 2.13 |
| factor(X25.CS2)=2 | 132 | 132 | 132 | 109 | 1.948 | 0.273 | 0.3 | 0.24 | 0.62 |

Created by EmpowerStats (www.empowerstats.com) and R on 2025-09-27
